# Supplementary material for: ATP synthase evolution on a cross-braced dated tree of life
Source: Nat Commun. 2023 Nov 17;14:7456. doi: 10.1038/s41467-023-42924-w (PMC10656485; doi:10.1038/s41467-023-42924-w)
Supplement: Supplementary file 3 — Description of Additional Supplementary Files [file 41467_2023_42924_MOESM3_ESM.pdf]

Description of Additional Supplementary Files

File Name: Supplementary Data 1
NCBI and GTDB taxonomic information for 350 Archaea, 350 Bacteria, and 100 Eukaryota (800 reference taxa) included in this study.

File Name: Supplementary Data 2
Annotation table of proteins in 800 reference taxa. Protein annotations were derived from several different databases (see Methods): KO = KEGG Orthology; COG = NCBI Clusters of Orthologous groups and Pfam.

File Name: Supplementary Data 3
List of COGs representing the F- and A/V-type ATP synthase subunits and three lipid biosynthesis genes (see Methods).

File Name: Supplementary Data 4
Count table of F- and A/V-type ATP synthase subunits represented by COG families (see Methods, Figure 1, Supplementary Figure 2, Supplementary Data 3) in 800 reference species. Includes quantification and curation of eukaryotic ATP synthase sequences flagged as putative bacterial contamination (see Methods).

File Name: Supplementary Data 5
Table summarizing presence of key metabolic organelles in 100 Eukaryotes sampled in this study. Information includes the presence of a true mitochondrion, a mitochondrion-related organelle (MRO), and/or plastid (primary, secondary, kleptoplast).

File Name: Supplementary Data 6
A summary table of the gene tree-species tree reconciliation results. Probability of each gene family being present at the root for different subunits, taxon sampling, substitution models, root positions and ancestral node. For each of these different conditions we infer either the presence or absence of the different subunits for LACA, LBCA and LUCA. AB = root between Archaea and Bacteria. Grac = root within Bacteria, sister to Gracilicutes. Euks = eukaryotes. C60 and C20 = the number of amino-acid replacement profiles in the mixture model for species tree estimation (see Methods). Node_number = corresponding node number on the species tree. PP = presence probability. LL = loglikelihood. SUM_LL = sum of all subunit loglikelihoods for that species tree and taxon sampling. OR1 = default origination rate parameters.

File Name: Supplementary Data 7
Ancestral sequence reconstruction states table. Inferred amino acid states at each position of each node in the combined ATP synthase protein phylogeny (Figure 3A, Supplementary Figure 10, see Methods).

File Name: Supplementary Data 8

Summary of key phylogenetic results from ATP synthase protein phylogenies (Supplementary Figures 4-10). All alignments, treefiles, and other IQ-TREE output files are available in our Zenodo data repository: 10.5281/zenodo.10012837)

File Name: Supplementary Data 9
Fossil calibration node assignments (i.e., calibration, node leaf/taxon assignment, age ranges, and probability masses) and braces (node leaf/taxon assignments) information applied to the Edited1, Edited2, and ATP synthase gene tree dating analyses.

File Name: Supplementary Data 10
Summarized age ranges for key speciation nodes in the species tree and ATP synthase gene tree dating analyses.

File Name: Supplementary Data 11
Taxonomic information and select metadata for 100 eukaryotic genomes/transcriptomes included in the study.

File Name: Supplementary Data 12
Summary check sheet of manual inspection of single gene trees inferred for 27 single-copy marker genes (Moody et al., 2022, *eLife* 11:e66695) used to generate the concatenated species phylogeny. Manual inspection included checks for domain monophyly, and the presence of paralogous sequences, contaminating sequences, and long-branch attraction (LBA). Marker gene distribution was used to filter genomes with poor marker gene coverage (65% threshold). All alignments, treefiles, and other IQ-TREE output files are available in our Zenodo data repository: 10.5281/zenodo.10012837.

File Name: Supplementary Data 13
Count table of 21 (of 27 original) single-copy marker genes (Supplementary Data 12) present on genomes of 800 reference taxa. Includes count and percentage distribution for eukaryotic nuclear, mitochondrial, and plastid homologs which were used for marker-gene presence cutoffs (see Methods).

File Name: Supplementary Data 14
Summary of key phylogenetic results of the concatenated species phylogenies (inferred using 21 single-copy marker genes, see Methods and Supplementary Data 12) including model selection, software versions, and taxon selections. All alignments, treefiles, and other IQ-TREE output files are available in our Zenodo data repository: 10.5281/zenodo.10012837.

File Name: Supplementary Data 15
Results of Approximately Unbiased (AU) test to assess the statistical significance of the two concatenated species phylogenies inferred in IQ-TREE2 v2.1.2 with the LG+C20+R+F and LG+C60+R+F models (see Methods). All alignments, treefiles, and other IQ-TREE output files are available in our Zenodo data repository: 10.5281/zenodo.10012837.

File Name: Supplementary Data 16
Summary of Ribosomal marker genes used to generate concatenated species tree containing mitochondrial and plastid eukaryotic homologs (nuclear homologs are those selected from the original concatenated species tree analysis, see Supplementary Data 12). Data includes lists of mitochondrial and plastid homologs for each representative COG marker gene corresponding to the 12 single-copy ribosomal marker genes. Mitochondrial and plastid genes are listed for each of the 12 single-copy ribosomal marker genes. All alignments, treefiles, and other IQ-TREE output files are available in our Zenodo data repository: 10.5281/zenodo.10012837.

File Name: Supplementary Data 17
Taxonomic mapping files used to generate the presence-absence summary plots (Figure 1, Figure 2, and Supplementary Figure 2). Archaea (n=350), Bacteria (n=350), and Eukaryotes (n=80, see Methods for filtering steps) used were assessed at the clade- or species-level defined by the collapsed and long concatenated species tree, respectively (Figure 1, Supplementary Figure 2, Supplementary Figure 20). 100 eukaryotes used to assess organelle presence were clustered based on eukaryotic supergroups defined in Burki et al., 2020 (Burki et al., 2020, *Trends Ecol. Evol.* 35, 43–55) (Figure 2).

File Name: Supplementary Data 18
Bracing file (json) for Edited1 species tree (see Methods; Supplementary Figures 12, 14, 15; Supplementary Data 10).

File Name: Supplementary Data 19
Bracing file (json) for Edited2 species tree (see Methods; Figure 5C; Supplementary Figures 13, 16; Supplementary Data 10).

File Name: Supplementary Data 20
Bracing file (json) for ATP synthase gene tree (see Methods; Figure 3A; Supplementary Figures 10, 18, 19; Supplementary Data 10).

File Name: Supplementary Data 21
Results of Approximately Unbiased (AU) test to assess the statistical significance of the topologies of the maximum-likelihood (ML) and two constrained ribosomal concatenated phylogenies used for dating (ML, Edited1, and Edited2). All alignments, treefiles, and other IQ-TREE output files are available in our Zenodo data repository: 10.5281/zenodo.10012837.
