# Supplementary material for: ATP synthase evolution on a cross-braced dated tree of life
Source: Nat Commun. 2023 Nov 17;14:7456. doi: 10.1038/s41467-023-42924-w (PMC10656485; doi:10.1038/s41467-023-42924-w)
Supplement: Supplementary file 5 — Reporting Summary [file 41467_2023_42924_MOESM5_ESM.pdf]

nature portfolio

Corresponding author(s): Anja Spang, Tom Williams

Last updated by author(s): Oct 17, 2023

Reporting Summary

Nature Portfolio wishes to improve the reproducibility of the work that we publish. This form provides structure for consistency and transparency in reporting. For further information on Nature Portfolio policies, see our Editorial Policies and the Editorial Policy Checklist.

Please do not complete any field with "not applicable" or n/a. Refer to the help text for what text to use if an item is not relevant to your study.
For final submission: please carefully check your responses for accuracy; you will not be able to make changes later.

Statistics

For all statistical analyses, confirm that the following items are present in the figure legend, table legend, main text, or Methods section.

n/a	Confirmed	
☐	☒	The exact sample size (*n*) for each experimental group/condition, given as a discrete number and unit of measurement
☒	☐	A statement on whether measurements were taken from distinct samples or whether the same sample was measured repeatedly
☐	☒	The statistical test(s) used AND whether they are one- or two-sided<br>*Only common tests should be described solely by name; describe more complex techniques in the Methods section.*
☐	☒	A description of all covariates tested
☐	☒	A description of any assumptions or corrections, such as tests of normality and adjustment for multiple comparisons
☐	☒	A full description of the statistical parameters including central tendency (e.g. means) or other basic estimates (e.g. regression coefficient) AND variation (e.g. standard deviation) or associated estimates of uncertainty (e.g. confidence intervals)
☐	☒	For null hypothesis testing, the test statistic (e.g. *F*, *t*, *r*) with confidence intervals, effect sizes, degrees of freedom and *P* value noted<br>*Give P values as exact values whenever suitable.*
☐	☒	For Bayesian analysis, information on the choice of priors and Markov chain Monte Carlo settings
☒	☐	For hierarchical and complex designs, identification of the appropriate level for tests and full reporting of outcomes
☒	☐	Estimates of effect sizes (e.g. Cohen's *d*, Pearson's *r*), indicating how they were calculated

Our web collection on statistics for biologists contains articles on many of the points above.

Software and code

Policy information about availability of computer code

Data collection	KEGG Automatic Annotation Server (KAAS, downloaded April 2019), https://www.genome.jp/tools/kofamkoala/<br>NCBI COG database (downloaded May 2020). https://ftp.ncbi.nih.gov/pub/COG/COG2020/data/<br>UniProt Knowledge Base (November 2019), https://www.uniprot.org/<br>Fossil information from publication as listed in Supplementary material<br>Eukaryotic transcriptomes as provided in our Zenodo data repository: https://doi.org/10.5281/zenodo.10012837
Data analysis	Mcmcdate: https://github.com/dschrempf/mcmc-date<br>Small custom scripts and workflows: https://doi.org/10.5281/zenodo.10012837<br>hmmsearch v3.lb2, http://hmmer.org/download.html<br>IQ-TREE v1.6.7, http://www.iqtree.org/#download<br>IQ-TREE2 v.2.1.2, http://www.iqtree.org/#download<br>BMGE v1.12, https://bioweb.pasteur.fr/packages/pack@BMGE@1.12<br>TRIMAL vl.2rev59, http://trimal.cgenomics.org/downloads<br>PhyloBayes-MPI (version 1.5), https://github.com/bayesiancook/pbmpi<br>MAFFT v7.407, https://mafft.cbrc.jp/alignment/software/<br>MAFFT v7.453, https://mafft.cbrc.jp/alignment/software/<br>CD-HIT v4.7, https://github.com/weizhongli/cdhit<br>DIAMOND v0.9.22.123, https://github.com/bbuchfink/diamond<br>catfasta2phyml.pl, https://github.com/nylan der /catfasta2phyml)<br>Replace_tree_names.pl, https://github.com/ndombrowski/Phylogeny_tutorial/tree/main/Input_files/5_required_Scripts<br>Jalview v2.10.5, https://www.jalview.org/download/

WebLogo3 v3.7.4, http://weblogo.threeplusone.com/
ALE v1.0, https://github.com/ssolo/ALE

For manuscripts utilizing custom algorithms or software that are central to the research but not yet described in published literature, software must be made available to editors and reviewers. We strongly encourage code deposition in a community repository (e.g. GitHub). See the Nature Portfolio guidelines for submitting code & software for further information.

Data

Policy information about availability of data

All manuscripts must include a data availability statement. This statement should provide the following information, where applicable:

- Accession codes, unique identifiers, or web links for publicly available datasets
- A description of any restrictions on data availability
- For clinical datasets or third party data, please ensure that the statement adheres to our policy

All genomic data of Archaea and Bacteria analyzed are available at NCBI (Supplementary Data 1), while all eukaryotic genomic/transcriptomic material is deposited in our Zenodo data repository: https://doi.org/10.5281/zenodo.10012837). Additional supplementary files including single gene tree analyses and concatenated phylogenies (i.e., sequence files, alignments, and treefiles) are deposited in our Zenodo data repository, https://doi.org/10.5281/zenodo.10012837. Databases used in this study are detailed as follows: ATP synthase Interpro domains were downloaded from Uniprot Knowledge Base (2019) [https://www.uniprot.org/], KO profiles downloaded from the KEGG Automatic Annotation Server in 2019 [https://www.genome.jp/tools/kofamkoala/], and the NCBI COG Database downloaded May 2020 [https://ftp.ncbi.nih.gov/pub/COG/COG2020/data/].

Research involving human participants, their data, or biological material

Policy information about studies with human participants or human data. See also policy information about sex, gender (identity/presentation), and sexual orientation and race, ethnicity and racism.

Reporting on sex and gender	n/a
Reporting on race, ethnicity, or other socially relevant groupings	n/a
Population characteristics	n/a
Recruitment	n/a
Ethics oversight	n/a

Note that full information on the approval of the study protocol must also be provided in the manuscript.

Field-specific reporting

Please select the one below that is the best fit for your research. If you are not sure, read the appropriate sections before making your selection.

☐ Life sciences ☐ Behavioural & social sciences ☒ Ecological, evolutionary & environmental sciences

For a reference copy of the document with all sections, see nature.com/documents/nr-reporting-summary-flat.pdf

Ecological, evolutionary & environmental sciences study design

All studies must disclose on these points even when the disclosure is negative.

Study description	In this study, we have analysed the presence of ATP synthase subunits across a representative set of archaea, bacteria and eukaryotes across the tree of life. Using phylogenetics approaches as well as ancestral genome and sequence reconstruction approaches, we have analysed the evolutionary history of the A and B subunits of the ATP synthase. Furthermore, for both the ATP synthase phylogeny as well as a newly inferred ribosomal phylogeny, we have estimated a timescale of the ATP synthase and species tree using a novel molecular cross-bracing approach.
Research sample	We have analysed genomes (and transcriptomes) of ca. 800 Archaea, Bacteria and Eukaryotes
Sampling strategy	We have estimated initial species trees (see methods), which were subsequently subsampled to retain a representative set of about 350 archaeal genomes and 350 bacteria genomes and 100 eukaryotic genomes/transcriptomes. For Archaea and Bacteria, we preferentially selected Type-strains were, while high quality metagenome assembled genome and single cell assembled genomes were selected based on completeness and contamination levels.
Data collection	We collected genomic data from Archaea, Bacteria and Eukaryotes at NCBI and selected genomes based on the strategy briefly described above. Please see our detailed methods section for additional details.

Timing and spatial scale	We have selected all genomes at the beginning of this project (2019).
Data exclusions	Our phylogenetic analyses could only be performed on a subset of taxa/genome currently deposited at NCBI due to computational limitations. We have tried to select the highest quality genomes from a taxonomically representative set reflecting the known phylogenetic diversity of Archaea, Bacteria and Eukaryotes.
Reproducibility	We have taken care to provide a detailed method section, supplementary information as well as a data repository, which provides access to our data and code and ensure reproducibility.
Randomization	Genomic data was selected based on highest quality and taxonomic distance.
Blinding	Our paper reports phylogenetic and molecular clock analyses. Data were selected based on best practices, for example ensuring representative taxon sampling. These are analyses of observational data and blinding is not part of the standard analysis protocol.

Did the study involve field work? ☐ Yes ☒ No

Reporting for specific materials, systems and methods

We require information from authors about some types of materials, experimental systems and methods used in many studies. Here, indicate whether each material, system or method listed is relevant to your study. If you are not sure if a list item applies to your research, read the appropriate section before selecting a response.

Materials & experimental systems

n/a	Involved in the study
☒	☐ Antibodies
☒	☐ Eukaryotic cell lines
☒	☐ Palaeontology and archaeology
☒	☐ Animals and other organisms
☒	☐ Clinical data
☒	☐ Dual use research of concern
☒	☐ Plants

Methods

n/a	Involved in the study
☒	☐ ChIP-seq
☒	☐ Flow cytometry
☒	☐ MRI-based neuroimaging

This checklist template is licensed under a Creative Commons Attribution 4.0 International License, which permits use, sharing, adaptation, distribution and reproduction in any medium or format, as long as you give appropriate credit to the original author(s) and the source, provide a link to the Creative Commons license, and indicate if changes were made. The images or other third party material in this article are included in the article's Creative Commons license, unless indicated otherwise in a credit line to the material. If material is not included in the article's Creative Commons license and your intended use is not permitted by statutory regulation or exceeds the permitted use, you will need to obtain permission directly from the copyright holder. To view a copy of this license, visit http://creativecommons.org/licenses/by/4.0/
